# Opportunities for renal genetic evaluation among pregnant patients with kidney disease

**DOI:** 10.1002/pmf2.70265

**Published:** 2026-02-27

**Authors:** Likhita Nandigam, Christian M. Parobek, Taylor Moseley, Ansley E. Davis, Stacie G. Denning, Mir Reza, Sai K. Saridey, Hennie A. Lombaard

**Affiliations:** ^1^ School of Medicine Baylor College of Medicine Houston Texas USA; ^2^ Department of Obstetrics and Gynecology Baylor College of Medicine Houston Texas USA; ^3^ Department of Molecular and Human Genetics Baylor College of Medicine Houston Texas USA; ^4^ Department of Pediatrics Division of Pediatric Nephrology Baylor College of Medicine Houston Texas USA; ^5^ Department of Internal Medicine Baylor College of Medicine Houston Texas USA

**Keywords:** genetic testing, health disparities, renal disease

## Abstract

**Objective:**

Chronic kidney disease (CKD) can significantly affect pregnancy management and outcomes. Although up to 40% of adults with renal disease have an identifiable genetic cause, most pregnant patients with CKD do not undergo genetic testing, hindering tailored management during and after pregnancy. We assessed which pregnant patients with CKD were more likely to be referred for and undergo genetic counseling and testing.

**Study design:**

This was an Institutional Review Board–approved retrospective cohort study of all patients who received prenatal care at Texas Children's Hospital between 2011 and 2024 and had a diagnosis of CKD or end‐stage renal disease (ESRD). Patients were identified using International Classification of Diseases, 10th Revision (ICD‐10) codes in the electronic medical record. Demographic, clinical, and genetic data were abstracted and overlaid with social vulnerability index data (2022 release). Bivariate analyses were used to assess associations between demographic and clinical factors and genetic evaluation.

**Results:**

A total of 103 pregnant patients had a diagnosis of CKD or ESRD. The median gestational age at entry to prenatal care was 8.6 weeks. While a majority of patients (64%) were referred to genetic counseling for any reason, only one in four (27%) referrals mentioned a renal indication. Even fewer patients (8%) underwent genetic testing before, during, or after pregnancy to determine the etiology of their chronic renal disease. Among demographic, socioeconomic, and clinical variables, only renal transplant and a family history of renal disease were associated with a recommendation for a renal genetic workup. Commercial insurance coverage was associated with completion of a renal genetic workup.

**Conclusions:**

The majority of pregnant patients with renal disease did not undergo renal genetic testing before, during, or after pregnancy. Commercial insurance coverage was the only demographic, socioeconomic, or clinical factor significantly associated with completion of a genetic workup. While genetic evaluation for renal disease among pregnant patients is associated with insurance status, it is underutilized across demographic and socioeconomic sectors and should be considered for pregnant patients with renal disease.

## 1 INTRODUCTION

Approximately 3% of pregnancies in the United States are complicated by chronic kidney disease (CKD) [1, 2]. Pregnancies with CKD have a 12‐fold increased risk of maternal and fetal complications, including preeclampsia, cesarean delivery, fetal growth restriction, prematurity, and stillbirth [3, 4, 5, 6]. These risks increase with progressive CKD stages [5]. Several studies have concluded that renal function for patients with CKD worsens during and after pregnancy, yet few guidelines exist on the optimization of CKD during pregnancy, despite the increase in prevalence of CKD over time [3, 7].

While the rising prevalence of diabetes and hypertension may contribute to increased rates of kidney disease in pregnancy, many women diagnosed with kidney disease have no other comorbidities [8]. Genetic causes of kidney disease are common, yet frequently underdiagnosed [9]. Over 600 genes and copy‐number variants have been linked to kidney disease, and a genetic etiology is identified in up to 50% of non‐diabetic childhood‐onset kidney disease and up to 30% of non‐diabetic adult‐onset kidney disease [9, 10, 11]. Identifying a genetic etiology for renal disease can guide management; [11, 12, 13] multiple studies highlight the benefits of securing a genetic diagnosis for CKD, including informing prognosis, changing therapy, and initiating tailored subspeciality care [11, 12, 13, 14, 15, 16, 17].

Recommendations pertaining to genetic testing for CKD have changed dramatically in recent years. For example, in 2012, the Kidney Disease: Improving Global Outcomes (KDIGO) Glomerulitis Work Group recommended against genetic testing for adults with focal segmental glomerulonephritis (FSGS) [18, 19]. As evidence for a molecular basis of renal disease accumulated over the following decade, the 2021 KDIGO practice guidelines were updated to allow for genetic testing in FSGS and to recommend genetic testing for familial or syndromic cases [18, 20]. Since that time, momentum has grown for genetic evaluation of adults with renal disease [1, 18]. The National Kidney Foundation published its first consensus guidelines for renal genetic testing in 2024, which recommended genetic testing for cases with FSGS, tubulopathies, steroid‐resistant nephrotic syndrome, atypical cystic kidney or liver disease, a relevant family history, prognostication, and family planning [21, 22].

Genetic testing for renal disease is more accessible than ever before. Renal‐specific panels are available from a number of commercial laboratories and are recommended as a first step to identifying a genetic etiology of kidney disease [18]. Furthermore, the falling cost of exome and genome sequencing should enable broader access to molecular answers in complex or syndromic cases. However, despite the availability of genetic testing for renal disease, several factors limit the proportion of patients who undergo genetic testing. Uninsured patients often present late to care with more severe renal disease, prolonging the time to molecular diagnosis [23, 24]. While some insurance companies cover genetic testing if a patient has an onset of disease under 40 years, they require proof of an extensive negative workup, which is both time and cost‐intensive [25]. CKD and end‐stage renal disease (ESRD) rates, as well as the risk of progression from CKD to ESRD, are disproportionately higher among black Americans as compared to white Americans [26]. Despite this, it is well documented that non‐White patients have a lower likelihood of undergoing genetic testing across varying diseases [23, 24, 27, 28].

At this time, there are no specific recommendations regarding genetic testing for renal disease in the context of pregnancy [1]. Limited literature exists on renal genetic testing during pregnancy; a retrospective chart review found that only two of 19 patients with CKD underwent genetic testing for CKD during pregnancy, both of which were due to fetal abnormality indications [29]. Overall, recommendations for genetic diagnosis and management of chronic renal disease during pregnancy are limited and amplify existing inequity [30].

This study sought to assess the drivers behind recommendations for genetic counseling and genetic workup for renal disease during pregnancy, as well as the rates of subsequent genetic testing and diagnosis. We predicted that patients with increased socioeconomic barriers would have lower rates of access to and completion of renal genetic testing.

## 2 METHODS

This was a retrospective chart review of pregnant patients with CKD or ESRD who received prenatal care at Texas Children's Hospital (TCH) between 2011 and 2024. The Baylor College of Medicine Institutional Review Board (IRB) approved this project (#H‐55646).

Female patients with a pregnancy episode and concurrent International Classification of Diseases, 10th Revision (ICD‐10) diagnosis of CKD or ESRD, as well as documentation of at least one prenatal care visit and delivery at TCH between 2011 and 2024, were included in this study. Pregnancies that resulted in termination or spontaneous abortion before 20 weeks of gestation were excluded due to the limited duration of prenatal care and the lower likelihood of a comprehensive genetic evaluation. In cases where a single patient had multiple eligible pregnancies, the most recent pregnancy was abstracted and included in this analysis.

For included patients, variables pertaining to demographics, medical history, prenatal care, and delivery course were extracted from the electronic medical record. The REDCap data management system was used for secure data collection and storage [31]. Detailed data pertaining to renal disease, workup, and management were abstracted from available records, including notes from visits outside of the pregnancy timeframe. Genetic counseling referrals, notes, and genetic testing laboratory results were reviewed in detail to verify genetic workup recommendation and completion during the antepartum, intrapartum, and postpartum period. A standardized framework was created and implemented to streamline data abstraction. Admission notes and discharge summaries provided insight into the indications for delivery as well as perinatal complications.

For each patient, the Social Vulnerability Index (SVI) was calculated based on the anonymized residential address during the pregnancy in question. SVI was developed by the Centers for Disease Control and Prevention (CDC) to stratify social vulnerability across geographical sectors [32]. The four primary categories include socioeconomic status, household characteristics, racial and ethnic minority status, and housing type and transportation.

To determine the strength of clinical associations between patient characteristics and outcomes of interest, bivariate analyses were used, including the Wilcoxon rank sum test, Pearson's Chi‐squared test, and Fisher's exact test as appropriate. Unadjusted and adjusted logistic regression analyses were performed to evaluate the association between selected clinical factors and the likelihood of receiving a recommendation for renal genetic testing. Odds ratios (ORs) and 95% confidence intervals (CIs) were estimated using binomial logistic regression. Analyses were performed in R and using the gtsummary package [33].

## 3 RESULTS

A total of 103 patients met the inclusion criteria; 93 patients had a diagnosis of CKD, and 10 patients had a diagnosis of ESRD. Most patients were diagnosed with renal disease before pregnancy (80%), though one in five were diagnosed during the included pregnancy (19%) and one was diagnosed in the postpartum period (1%). The population was diverse, including both Hispanic and non‐Hispanic individuals (44% vs. 56%, respectively), and 42% identified as non‐White (Table 1). The majority of patients utilized healthcare through public insurance (Medicare or Medicaid) or self‐pay (58%) as opposed to commercial insurance (43%). Despite varying insurance coverage, the average gestational age at entry to prenatal care remained in the first trimester, at eight weeks of gestation. Almost all patients were diagnosed with a prepregnancy comorbidity aside from renal disease (88%), including diabetes, hypertension, or autoimmune disease. Nearly half (44%) required dialysis before, during, or after pregnancy, and the majority of patients (52%) were induced for delivery due to a direct renal or hypertensive indication.

**TABLE 1:** Baseline demographic and clinical characteristics.

Variable	*N* = 103^a^
Maternal age at delivery (years)	31 (27, 36)
GA at entry to care (weeks)	8.6 (7.0, 11.4)
Hispanic ethnicity	45 (44%)
Race	
Asian	4 (4.0%)
Black	38 (38%)
White	58 (58%)
Preferred language: English	97 (94%)
Commercial insurance	44 (43%)
Body mass index (kg/m^2^)	28 (23, 34)
IVF pregnancy	4 (3.9%)
Nulliparity	40 (39%)
Renal disease diagnosed pre‐pregnancy	82 (80%)
Pre‐pregnancy comorbidity (DM, HTN, AI)	91 (88%)
Dialysis before, during, or after pregnancy	45 (44%)
Renal transplant before pregnancy	11 (11%)
Indication for delivery: HTN or renal disease	54 (52%)

Abbreviations: AI, autoimmune disease; DM, diabetes mellitus; GA, gestational age; HTN, hypertension; IVF, in vitro fertilization.

^a^ Median (Q1, Q3); *n* (%).

We explored the demographic and clinical factors associated with a recommendation for genetic testing. There was no association between demographic or socioeconomic factors, including race, insurance, or SVI, with a recommendation for renal genetic workup. Only two clinical factors were associated with a recommendation for genetic workup: prepregnancy renal transplant and a positive family history of an affected first‐degree relative with kidney disease (Table 2). Commercial insurance coverage was the sole factor associated with completion of renal genetic testing (Table 2).

**TABLE 2:** Demographic and clinical associations with recommendation for and completion of renal genetic workup among pregnant patients.

	Recommended			Completed		
Characteristic	Yes *N* = 19^a^	No *N* = 84^a^	*p* value^b^	Yes *N* = 8^a^	No *N* = 11^a^	*p* value^c^
Maternal age at delivery (years)	31 (25, 36)	31 (27, 35)	0.6	34 (24, 37)	27 (25, 36)	0.8
GA at entry to care (weeks)	8.1 (6.0, 13.1)	8.6 (7.4, 10.9)	0.7	7 (6, 12)	9 (6, 13)	0.6
Hispanic ethnicity	9 (47%)	36 (43%)	0.7	5 (63%)	5 (45%)	0.6
Race			0.7			0.2
Asian	1 (5.6%)	3 (3.7%)		1 (13%)	0 (0%)	
Black	6 (33%)	32 (39%)		1 (13%)	5 (45%)	
White	11 (61%)	47 (57%)		6 (75%)	6 (55%)	
Commercial insurance	9 (47%)	35 (42%)	0.7	7 (88%)	2 (18%)	0.005
Social Vulnerability Index (SVI)	0.71 (0.51, 0.93)	0.73 (0.54, 0.88)	>0.9	0.62 (0.35, 0.82)	0.82 (0.57, 0.93)	0.4
SVI socioeconomic status	0.79 (0.42, 0.93)	0.77 (0.56, 0.91)	>0.9	0.59 (0.40, 0.89)	0.87 (0.60, 0.93)	0.4
SVI household composition & disability	0.82 (0.50, 0.89)	0.71 (0.46, 0.90)	0.5	0.71 (0.51, 0.88)	0.76 (0.42, 0.89)	0.8
SVI minority status & language	0.88 (0.71, 0.94)	0.84 (0.74, 0.94)	0.8	0.85 (0.70, 0.94)	0.88 (0.75, 0.95)	0.7
SVI housing type & transportation	0.47 (0.23, 0.67)	0.49 (0.32, 0.70)	0.6	0.27 (0.13, 0.66)	0.47 (0.36, 0.67)	0.3
Nulliparity	10 (53%)	30 (36%)	0.2	6 (75%)	5 (45%)	0.4
IVF pregnancy	2 (11%)	2 (2.4%)	0.2	2 (25%)	0 (0%)	0.2
Body mass index (kg/m^2^)	29 (22, 36)	28 (23, 33)	0.9	34 (26, 36)	26 (20, 31)	0.2
Affected first‐degree relative	8 (42%)	14 (17%)	0.027	3 (38%)	5 (45%)	>0.9
Prepregnancy renal disease diagnosis	17 (89%)	65 (77%)	0.3	8 (100%)	9 (82%)	0.5
Prepregnancy hypertension	15 (79%)	62 (74%)	0.8	6 (75%)	10 (91%)	0.5
Prepregnancy diabetes	2 (11%)	27 (32%)	0.058	0 (0%)	2 (18%)	0.5
Dialysis before, during, or after pregnancy	9 (47%)	36 (43%)	0.7	4 (50%)	6 (55%)	>0.9
Renal transplant before pregnancy	5 (26%)	6 (7.2%)	0.030	3 (38%)	2 (18%)	0.6
ICU admission during pregnancy	5 (26%)	18 (21%)	0.8	3 (38%)	2 (18%)	0.6

*Note*: Genetic workup recommendations included all phases of care, within and outside of pregnancy.

Abbreviations: GA, gestational age; IVF, in vitro fertilization.

^a^ Median (Q1, Q3); *n* (%).

^b^ Wilcoxon rank sum test; Pearson's Chi‐squared test; Fisher's exact test.

^c^ Wilcoxon rank sum test; Fisher's exact test; Wilcoxon rank sum exact test.

Based on known associations between demographic and economic disparities in kidney disease, we specifically investigated the impact of race and insurance status on the likelihood of recommendation for and completion of genetic testing. From this analysis, it was determined that Black patients had significantly higher rates of prepregnancy hypertension (95% vs. 63%, *p* < 0.001) and post‐pregnancy dialysis (45% vs. 25%, *p* = 0.035) as compared to non‐Black patients (Table S1). However, Black race was not associated with a higher rate of genetic testing referrals or completed workup. The data also suggest that non‐Black patients were more likely to have their genetic counseling visit address CKD (*p* = 0.05). When patients were compared by use of public or self‐pay insurance as compared to commercial insurance for prenatal care, the latter was significantly associated with completion of genetic testing (Table S2). Patients without commercial insurance had later entry to prenatal care (10.1 vs. 7.6 weeks, *p* < 0.001) than those with commercial insurance (Table S2). Finally, while limited power prevents conclusive analysis, there is a notable trend of increased need for dialysis across all pregnancy stages among those lacking private insurance (*p* = 0.08).

**FIGURE 1:**
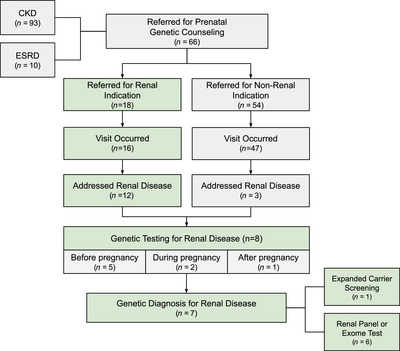
Schematic of pregnant patients with renal disease who underwent genetic testing. CKD, chronic kidney disease; ESRD, end‐stage renal disease.

In our cohort of pregnant patients with kidney disease, only a minority were recommended for or completed a genetic workup for renal disease. Of 103 total patients, a genetic workup was recommended by a physician or genetic counselor in 19 cases (18%). Completion of molecular renal genetic testing before, during, or after pregnancy was documented for only eight patients (8%). A schematic of the genetic referral and testing rates is shown in Figure 1. Less than one third of patients referred to genetic counseling had renal disease listed as an indication for referral. Less than one sixth of patients who were referred to genetic counseling for any reason participated in a genetic counseling session that addressed their renal disease.

A temporal analysis was performed to determine whether patients with an estimated due date (EDD) on or after January 1, 2022 experienced different rates of recommendation for or completion of genetic testing. This date was selected due to the publication of KDIGO practice guidelines in 2021, which recommended renal genetic testing in some circumstances. In this subanalysis, 13 of 52 (25%) patients with EDD on or after January 1, 2022, received a recommendation for renal genetic testing compared with 6 of 51 (12%) in the pre‐2022 group (*p* = 0.083). Of those who received a recommendation for genetic testing, seven of 13 (54%) patients with EDD on or after January 1, 2022 underwent genetic testing compared with one of six (17%) in the pre‐2022 group (*p* = 0.2).

## 4 DISCUSSION

This study highlights the low rates of genetic evaluation and testing among pregnant patients with CKD. Family history of renal disease and personal history of renal transplant were the only two demographic and clinical variables associated with a recommendation for renal genetic testing. Furthermore, commercial insurance coverage was correlated with completion of renal genetic testing. When further stratified by race and insurance, two known barriers to genetic testing, black race was associated with higher pre‐pregnancy hypertension rates and trended toward fewer genetics discussions that addressed renal disease [28, 34].

The high drop‐off rates between pregnant patients known to have renal disease, those referred for genetic testing for renal disease, and those who undergo genetic testing for renal disease emphasize a key gap in prenatal care for all renal patients, regardless of socioeconomic factors or access to clinical care. Provider discomfort, competing priorities in prenatal management, limited genetic testing availability, and inconsistent referral workflow may all contribute to the discordance between high disease burden and low testing rates. To date, prenatal care providers and genetic counselors have not been surveyed to assess reasons for low referral and counseling rates. A survey of nephrologists found that perceived barriers of cost, poor availability, and lack of ease contributed to low referral rates for genetic testing for CKD patients [35].

**BOX 1: Renal genetic testing recommendations during pregnancy**

*Testing for renal genetic conditions in high‐risk individuals during pregnancy may increase access to genetic care, improve perinatal and postnatal kidney care, and enable informed family planning*.

**Consider maternal renal genetic testing when one or more of the following features are present [** 1, 9]

1. Early or atypical onset of kidney disease
  - CKD in childhood or early adulthood (e.g., before 25–50 years) [38, 45]
  - CKD presenting earlier than expected for common acquired etiologies
2. CKD of unknown or unclear etiology
  - CKD or kidney failure without a clear cause
  - Situations in which kidney biopsy is impractical or unlikely to be informative
3. Family history suggestive of inherited kidney disease
  - Family history of CKD, dialysis, kidney transplantation, or unexplained kidney failure
  - Known or suspected inherited kidney disorder in relatives
4. Consanguinity or suspected autosomal recessive inheritance
  - Parental consanguinity
  - Multiple affected siblings or recurrence consistent with recessive disease
5. Syndromic, dysmorphic, or extrarenal manifestations
  - Multisystem involvement
  - Dysmorphic features or a recognizable clinical syndrome
6. Specific renal phenotypes enriched for monogenic disease (e.g., cystic disease, steroid‐resistant nephrotic syndrome, chronic glomerular hematuria or proteinuria, or focal segmental glomerulosclerosis with unclear cause)
  - Potential for therapeutic, prognostic, or management impact
  - Possibility of identifying a condition amenable to targeted therapy
7. Results that would alter prognosis, surveillance, or family counseling

**Maternal renal genetic testing options**

*Some commercial laboratories that offer reproductive carrier screening also offer renal genetic panels. Turnaround times for listed tests range from 2 to 6 weeks*.

1. Renal genetic panels: Consider for patients with a well‐defined or limited renal‐disease phenotype
  - Targeted renal panels: Include a few to dozens of genes associated with a specific renal phenotype such as polycystic kidney disease or Alport syndrome
  - Comprehensive renal panels: Include hundreds of genes associated with syndromic and non‐syndromic renal disease
2. Broad sequencing: Consider for patients with multisystem involvement suggestive of a complex genetic syndrome or if a genetic etiology is still suspected after unrevealing panel testing
  - Exome sequencing: Utilizes target capture prior to sequencing
  - Genome sequencing: No target capture prior to sequencing

*Pathogenic variants in PKD1 account for the majority of autosomal dominant polycystic kidney disease. Due to high sequence homology between PKD1 and pseudogenes, genotyping is technically challenging. If polycystic kidney disease is suspected clinically, ensure that the genetic test selected is validated for PKD1*.

Studies highlight a diagnostic yield of up to 37% for the genetic basis to CKD [13, 17, 36, 37, 38]. Receiving a genetic diagnosis may alter care in up to 90% of cases, with one third of patients benefiting from tailored treatment plans [17]. Studies have also identified disease reclassification rates ranging from 22% to 70% for patients with a diagnostic variant [36, 39, 40]. Examples include reclassification from FSGS to Alport syndrome, diabetic nephropathy to *TBX6*‐related CAKUT, and unspecified glomerular disease to *WDR19*‐related nephronophthisis. Because early genetic testing for renal disease can improve care and reduce overall medical costs by over 40%, prenatal care providers should be aware of genetic contributors to renal disease [16]. Furthermore, as CKD is associated with infertility, genetic testing should be considered at pre‐conception visits for patients with renal disease [41, 42]. Preimplantation genetic testing (PGT) for monogenic causes of renal disease would additionally inform and reduce the generational impacts of kidney disease with genetic etiologies [42]. It is well established that genetic‐testing rates for renal disease are lower among patients with socioeconomic barriers, and our study identified this pattern among pregnant patients with renal disease [26, 30]. Patients with socioeconomic barriers often present to care at more severe stages of CKD and require more urgent interventions [25]. Delayed entry to care and greater disease burden for lower‐income and publicly insured patients limits timely genetic testing evaluation. Genetic testing should be prioritized at earlier stages of CKD for all patients, and in early stages of pregnancy, to optimize renal management both during and after pregnancy. As pregnant patients have improved access to care and expanded insurance coverage, pregnancy presents a critical and accessible opportunity to undergo a genetic workup for renal disease [41, 43]. Yet, despite the public insurance coverage provided to pregnant patients, our study highlights limitations in public insurance in providing comprehensive genetic testing.

Whether pregnancy is the ideal time for renal genetic testing warrants careful consideration. Perceived and real barriers do exist to deploying diagnostic renal genetic testing in the prenatal period. First, obstetricians and prenatal genetic counselors may not feel comfortable with ordering or interpreting this type of genetic test. Furthermore, genetic testing not thought to be directly relevant to pregnancy management may cause an unwelcome increase in the emotional and clinical load of an already complex pregnancy (i.e., one complicated by poor renal function). Finally, a positive genetic result could introduce uncertainty regarding whether and when the fetus should be tested. On the other hand, pregnancy represents a unique opportunity as patients present regularly for medical care and have insurance coverage. Delaying genetic testing for this population jeopardizes the opportunity to identify a genetic diagnosis, as many lose insurance coverage after pregnancy. Finally, securing a genetic diagnosis may in some cases change pregnancy management, as in the case of polycystic kidney disease, where pregnancy heightens the risk of intracranial aneurysms [44]. For these reasons, we advocate that genetic testing be considered for pregnant patients with renal disease. Box 1 provides a framework for obstetrical care providers to evaluate a patient's candidacy for renal genetic testing and to understand testing options.

Strengths of this study include the inclusion of a demographically diverse cohort receiving care at a large referral center for maternity care [46]. Furthermore, this cohort comprised a broad range of renal severity and pathology, including patients on dialysis and those who received a renal transplant. Patients receiving prenatal care at our hospital also have widespread access to genetic counseling services regardless of insurance type. This, combined with the presence of a renal specialty clinic for pregnant patients as well as a renal genetics clinic, provides a platform for increased access to a renal genetic evaluation.

This study is not without limitations. Our diverse cohort receiving care at a single tertiary medical center may not reflect populations throughout the country, particularly in rural areas with limited access to prenatal care or genetic counseling. Furthermore, the pregnancies included in this analysis spanned 14 years, during which time there were significant advances in understanding the genetic basis for renal disease, as well as in the accessibility of renal genetic testing. Some patients may have been assigned a presumptive genetic diagnosis (e.g., polycystic kidney disease) without formal genetic evaluation or testing. Presumptive genetic diagnoses were not considered in this study. Furthermore, this study was not powered to test the likelihood of genetic testing among subpopulations such as racial minorities or those with public insurance. The low number of patients who underwent renal genetic testing limits extrapolations of statistically significant findings and highlights the need for further investigation in larger cohorts. However, this does not lessen the relevance of the primary finding that there is a broad‐based lack of renal genetic evaluation for pregnant patients with kidney disease.

While this study was not powered for robust temporal analyses, it should be noted that the proportion of patients with a due date from 2022 onward who received a recommendation for or completion of genetic testing was dramatically higher than the proportion of patients with a due date before 2022. Although this difference was not significant, it suggests that awareness and uptake of renal genetic testing have increased over time. Multicenter contemporary studies of pregnant patients with renal disease could provide the sample size needed to determine whether access to diagnostic renal genetic testing is improving.

Pregnancy presents a unique opportunity for identifying, working up, and proactively managing chronic renal disease. Despite this, and despite the known benefits and accessibility of genetic evaluation, only a small minority of pregnant patients with renal disease undergo a genetic workup. The low genetic testing rate likely stems from a lack of awareness among both obstetricians and genetic counselors regarding the odds and benefits of a positive genetic diagnosis. As the costs of genetic testing continue to decline and the discovery of an etiology becomes more feasible, prenatal care providers should weigh the perceived barriers to genetic testing against the tangible benefits for pregnancy management among patients with renal disease [16].

## CONFLICT OF INTEREST STATEMENT

The authors declare no conflicts of interest.

## FUNDING INFORMATION

The authors received no specific funding for this work.

## ETHICS STATEMENT

Minimal to no harm was placed on patients and information remained protected and private during this study.

## Supporting information



Supporting Information

## Data Availability

The data that support the findings of this study are available on request from the corresponding author. The data are not publicly available due to privacy or ethical restrictions.

## References

[1] Stevens, P. E. , S. B. Ahmed , J. J. Carrero , B. Foster , A. Francis , R. K. Hall , W. G. Herrington , et al. 2024. “KDIGO 2024 Clinical Practice Guideline for the Evaluation and Management of Chronic Kidney Disease.” Kidney International 105(4): S117–S314. 10.1016/j.kint.2023.10.018. 38490803

[2] Chu, C. D. , N. R. Powe , C. E. McCulloch , D. C. Crews , Y. Han , J. L. Bragg‐Gresham , R. Saran , et al. 2021. “Trends in Chronic Kidney Disease Care in the US by Race and Ethnicity, 2012–2019.” JAMA Network Open 4(9): e2127014. 10.1001/jamanetworkopen.2021.27014. 34570204 PMC8477264

[3] Gouveia, I. F. , J. R. Silva , C. Santos , and C. Carvalho . 2021. “Maternal and Fetal Outcomes of Pregnancy in Chronic Kidney Disease: Diagnostic Challenges, Surveillance and Treatment Throughout the Spectrum of Kidney Disease.” Brazilian Journal of Nephrology 43(1): 88–102. 10.1590/2175-8239-JBN-2020-0055. 33460427 PMC8061969

[4] Piccoli, G. B. , G. Cabiddu , R. Attini , F. N. Vigotti , S. Maxia , N. Lepori , M. Tuveri , et al. 2015. “Risk of Adverse Pregnancy Outcomes in Women With CKD.” Journal of the American Society of Nephrology 26(8): 2011–2022. 10.1681/ASN.2014050459. 25766536 PMC4520166

[5] Jeyaraman, D. , B. Walters , K. Bramham , R. Fish , M. Lambie , and P. Wu . 2024. “Adverse Pregnancy Outcomes in Pregnant Women With Chronic Kidney Disease: A Systematic Review and Meta‐Analysis.” BJOG 131(10): 1331–1340. 10.1111/1471-0528.17807. 38488268

[6] Khalaf, S. Y. A. , É. J. O'Reilly , F. P. McCarthy , M. Kublickas , K. Kublickiene , and A. S. Khashan . 2021. “Pregnancy Outcomes in Women With Chronic Kidney Disease and Chronic Hypertension: A National Cohort Study.” American Journal of Obstetrics and Gynecology 225(3): 298.e1–20. 10.1016/j.ajog.2021.03.045. 33823152

[7] Hui, D. , and M. A. Hladunewich . 2019. “Chronic Kidney Disease and Pregnancy.” Obstetrics and Gynecology 133(6): 1182. 10.1097/AOG.0000000000003256. 31135733

[8] Kawakita, T. , M. Hayasaka , L. Robbins , J. Martins , and G. Saade . 2025. “Association Between the Social Vulnerability Index and Adverse Pregnancy Outcomes.” Obstetrics and Gynecology 145(5): 503–510. 10.1097/AOG.0000000000005890. 40146994 PMC11999087

[9] Vivante, A. 2024. “Genetics of Chronic Kidney Disease.” New England Journal of Medicine 391(7): 627–639. 10.1056/NEJMra2308577. 39141855

[10] KDIGO Conference Participants . 2022. “Genetics in Chronic Kidney Disease: Conclusions From a Kidney Disease: Improving Global Outcomes (KDIGO) Controversies Conference.” Kidney International 101(6): 1126–1141. 10.1016/j.kint.2022.03.019. 35460632 PMC9922534

[11] Elliott, M. D. , H. M. Rasouly , and A. G. Gharavi . 2023. “Genetics of Kidney Disease: The Unexpected Role of Rare Disorders.” Annual Review of Medicine 74: 353–367. 10.1146/annurev-med-042921-101813. 36375470

[12] Wuttke, M. , Y. Li , M. Li , K. B. Sieber , M. F. Feitosa , M. Gorski , A. Tin , et al. 2019. “A Catalog of Genetic Loci Associated With Kidney Function From Analyses of a Million Individuals.” Nature Genetics 51(6): 957–972. 10.1038/s41588-019-0407-x. 31152163 PMC6698888

[13] Groopman, E. E. , M. Marasa , S. Cameron‐Christie , S. Petrovski , V. S. Aggarwal , H. Milo‐Rasouly , Y. Li , et al. 2019. “Diagnostic Utility of Exome Sequencing for Kidney Disease.” New England Journal of Medicine 380(2): 142–151. 10.1056/NEJMoa1806891. 30586318 PMC6510541

[14] Thomas, C. P. , M. E. Freese , A. Ounda , J. G. Jetton , M. Holida , L. Noureddine , and R. J. Smith 2020. “Initial Experience From a Renal Genetics Clinic Demonstrates a Distinct Role in Patient Management.” Genetics in Medicine 22(6): 1025–1035. 10.1038/s41436-020-0772-y. 32203225 PMC7272321

[15] Tan, X. Y. , C. Borden , M. B. Roberts , S. Mazzola , Q. K. Tan , R. Fatica , J. Simon , et al. 2023. “Renal Genetics Clinic: 3‐Year Experience in the Cleveland Clinic.” Kidney Medicine 5(2): 100585. 10.1016/j.xkme.2022.100585. 36712315 PMC9874141

[16] Becherucci, F. , S. Landini , V. Palazzo , L. Cirillo , V. Raglianti , G. Lugli , L. Tiberi , et al. 2023. “A Clinical Workflow for Cost‐Saving High‐Rate Diagnosis of Genetic Kidney Diseases.” Journal of the American Society of Nephrology 34(4): 706–720. 10.1681/ASN.0000000000000076. 36753701 PMC10103218

[17] Dahl, N. K. , M. S. Bloom , F. T. Chebib , D. Clark , M. Westemeyer , S. Jandeska , Z. Zhang , et al. 2023. “The Clinical Utility of Genetic Testing in the Diagnosis and Management of Adults With Chronic Kidney Disease.” Journal of the American Society of Nephrology 34(12): 2039. 10.1681/ASN.0000000000000249. 37794564 PMC10703084

[18] Beck, L. H. , I. Ayoub , D. Caster , M. J. Choi , J. Cobb , D. Geetha , M. N. Rheault , et al. 2023. “KDOQI US Commentary on the 2021 KDIGO Clinical Practice Guideline for the Management of Glomerular Diseases.” American Journal of Kidney Diseases 82(2): 121–175. 10.1053/j.ajkd.2023.02.003. 37341661

[19] 2012. “Abstract.” Kidney International Supplements 2(2): 142. 10.1038/kisup.2012.12.

[20] Rovin, B. H. , S. G. Adler , J. Barratt , F. Bridoux , K. A. Burdge , T. M. Chan , H. T. Cook , et al. 2021. “KDIGO 2021 Clinical Practice Guideline for the Management of Glomerular Diseases.” Kidney International 100(4): S1–S276. 10.1016/j.kint.2021.05.021. 34556256

[21] National Kidney Foundation . NKF KDOQI Clinical Practice Guidelines. Accessed August 12, 2025. https://www.kidney.org/professionals/kdoqi.

[22] Torres, V. E. , C. Ahn , T. R. M. Barten , G. Brosnahan , M. A. Cadnapaphornchai , A. B. Chapman , E. Cornec‐Le Gall , et al. 2025. “KDIGO 2025 Clinical Practice Guideline for the Evaluation, Management, and Treatment of Autosomal Dominant Polycystic Kidney Disease (ADPKD): Executive Summary.” Kidney International 107(2): 234–254. 10.1016/j.kint.2024.07.010. 39848746

[23] Iorember, F. M. , and O. F. Bamgbola . 2022. “Structural Inequities and Barriers to Accessing Kidney Healthcare Services in the United States: A Focus on Uninsured and Undocumented Children and Young Adults.” Frontiers in Pediatrics 10: 833611. 10.3389/fped.2022.833611. 35450110 PMC9016185

[24] Hall, Y. N. , R. A. Rodriguez , E. J. Boyko , G. M. Chertow , and A. M. O'Hare . 2009. “Characteristics of Uninsured Americans With Chronic Kidney Disease.” Journal of General Internal Medicine 24(8): 917–922. 10.1007/s11606-009-1028-3. 19506974 PMC2710472

[25] Ghazi, L. , T. L. Osypuk , R. F. MacLehose , R. V. Luepker , and P. E. Drawz . 2021. “Neighborhood Socioeconomic Status, Health Insurance, and CKD Prevalence: Findings From a Large Health Care System.” Kidney Medicine 3(4): 555–564.e1. 10.1016/j.xkme.2021.03.008. 34401723 PMC8350830

[26] Norton, J. M. , M. M. Moxey‐Mims , P. W. Eggers , A. S. Narva , R. A. Star , P. L. Kimmel , and G. P. Rodgers 2016. “Social Determinants of Racial Disparities in CKD.” Journal of the American Society of Nephrology 27(9): 2576–2595. 10.1681/ASN.2016010027. 27178804 PMC5004663

[27] Canedo, J. R. , S. T. Miller , H. F. Myers , and M. Sanderson . 2019. “Racial and Ethnic Differences in Knowledge and Attitudes about Genetic Testing in the U.S.: Systematic Review.” Journal of Genetic Counseling 28(3): 587–601. 10.1002/jgc4.1078. 30663831 PMC8081647

[28] Dusic, E. J. , T. Theoryn , C. Wang , E. M. Swisher , and D. J. Bowen . 2022. “Barriers, Interventions, and Recommendations: Improving the Genetic Testing Landscape.” Frontiers in Digital Health 4: 961128. 10.3389/fdgth.2022.961128. 36386046 PMC9665160

[29] Marchão, N. , J. O. Da Costa , N. Peres , I. Godinho , S. Jorge , M. Rodrigues , S. Gonçalves , et al. 2024. “#2095 Genetic Kidney Disease In Pregnancy—Experience of Tertiary Referral Hospital.” Nephrology Dialysis Transplantation 39(Supplement_1): gfae069–1325–2095. 10.1093/ndt/gfae069.1325.

[30] Crews, D. C. , A. K. Bello , and G. Saadi . 2019. “Burden, Access, and Disparities in Kidney Disease.” Kidney International Reports 4(3): 372–379. 10.1016/j.ekir.2019.01.011. 30899864 PMC6409385

[31] Harris, P. A. , R. Taylor , R. Thielke , J. Payne , N. Gonzalez , and J. G. Conde . 2009. “Research Electronic Data Capture (REDCap)—A Metadata‐Driven Methodology and Workflow Process for Providing Translational Research Informatics Support.” Journal of Biomedical Informatics 42(2): 377–381. 10.1016/j.jbi.2008.08.010. 18929686 PMC2700030

[32] CDC . “Social Vulnerability Index.” Place and Health—Geospatial Research, Analysis, and Services Program (GRASP). October 22, 2024. Accessed September 3, 2025. https://www.atsdr.cdc.gov/place‐health/php/svi/index.html.

[33] Sjoberg, D. D. , K. Whiting , M. Curry , J. A. Lavery , and J. Larmarange . 2021. “Reproducible Summary Tables With the gtsummary Package.” R Journal 13(1): 570–580. 10.32614/RJ-2021-053.

[34] Rodriguez, N. J. , C. Ricker , E. M. Stoffel , and S. Syngal . 2023. “Barriers and Facilitators to Genetic Education, Risk Assessment, and Testing: Considerations on Advancing Equitable Genetics Care.” Gastroenterology 164(1): 5–8. 10.1053/j.gastro.2022.11.021. 36529467 PMC11009722

[35] Mrug, M. , M. S. Bloom , C. Seto , M. Malhotra , H. Tabriziani , P. Gauthier , V. Sidlow , et al. 2021. “Genetic Testing for Chronic Kidney Diseases: Clinical Utility and Barriers Perceived by Nephrologists.” Kidney Medicine 3(6): 1050–1056. 10.1016/j.xkme.2021.08.006. 34939014 PMC8664736

[36] Connaughton, D. M. , C. Kennedy , S. Shril , N. Mann , S. L. Murray , P. A. Williams , E. Conlon , et al. 2019. “Monogenic Causes of Chronic Kidney Disease in Adults.” Kidney International 95(4): 914–928. 10.1016/j.kint.2018.10.031. 30773290 PMC6431580

[37] Snoek, R. , R. H. van Jaarsveld , T. Q. Nguyen , E. D. J. Peters , M. G. Elferink , R. F. Ernst , M. B. Rookmaaker , et al. 2022. “Genetics‐First Approach Improves Diagnostics of ESKD Patients <50 Years Old.” Nephrology Dialysis Transplantation 37(2): 349–357. 10.1093/ndt/gfaa363. 33306124

[38] Claus, L. R. , R. Snoek , N. Knoers,, and A. M. van Eerde . 2022. “Review of Genetic Testing in Kidney Disease Patients: Diagnostic Yield of Single Nucleotide Variants and Copy Number Variations Evaluated Across and Within Kidney Phenotype Groups.” American Journal of Medical Genetics Part C: Seminars in Medical Genetics 190(3): 358–376. 10.1002/ajmg.c.31995. 36161467 PMC9828643

[39] Lata, S. , M. Marasa , Y. Li , D. A. Fasel , E. Groopman , V. Jobanputra , H. Rasouly , et al. 2018. “Whole‐Exome Sequencing in Adults With Chronic Kidney Disease: A Pilot Study.” Annals of Internal Medicine 168(2): 100–109. 10.7326/M17-1319. 29204651 PMC5947852

[40] Shlomovitz, O. , D. Atias‐Varon , D. Yagel , O. Barel , H. Shasha‐Lavsky , K. Skorecki , A. Eliyahu , et al. 2024. “Genetic Markers Among the Israeli Druze Minority Population With End‐Stage Kidney Disease.” American Journal of Kidney Diseases 83(2): 183–195. 10.1053/j.ajkd.2023.06.006. 37717846

[41] Delgado, A. , J. Schulkin , and C. J. Macri . 2022. “Prenatal Genetic Screening and Diagnostic Testing: Assessing Patients' Knowledge, Clinical Experiences, and Utilized Resources in Comparison to Provider's Perceptions.” AJP Reports 12(1): e27–e32. 10.1055/s-0041-1742236. 35141032 PMC8816620

[42] Knoers, N. 2025. “Prenatal and Preimplantation Genetic Testing for Monogenic Kidney Disorders.” Kidney International 107(2): 255–261. 10.1016/j.kint.2024.06.031. 39477068

[43] Gregory, D. S. , V. Wu , and P. Tuladhar . 2018. “The Pregnant Patient: Managing Common Acute Medical Problems.” American Family Physician 98(9): 595–602. 30325641

[44] Al Sayyab, M. , and A. Chapman . 2023. “Pregnancy in Autosomal Dominant Polycystic Kidney Disease.” Advances Kidney Disease Health 30(5): 454–460. 10.1053/j.akdh.2023.10.006. 38032583

[45] Vivante, A. , and F. Hildebrandt . 2016. “Exome Sequencing Frequently Reveals the Cause of Early‐Onset Chronic Kidney Disease.” Nature Reviews Nephrology 12(3): 133–146. 10.1038/nrneph.2015.205. 26750453 PMC5202482

[46] Texas Children's . The Difference Is Life Changing. Accessed September 13, 2025. https://www.texaschildrens.org/.

[47] Mallawaarachchi, A. C. , Y. Hort , M. J. Cowley , M. J. McCabe , A. Minoche , M. E. Dinger , J. Shine , and T. J. Furlong 2016. “Whole‐Genome Sequencing Overcomes Pseudogene Homology to Diagnose Autosomal Dominant Polycystic Kidney Disease.” European Journal of Human Genetics 24(11): 1584–1590. 10.1038/ejhg.2016.48. 27165007 PMC5110048

