# Supplementary material for: Opportunities for renal genetic evaluation among pregnant patients with kidney disease
Source: Pregnancy (Hoboken). 2026 Feb 27;2(2):e70265. doi: 10.1002/pmf2.70265 (PMC13344408; doi:10.1002/pmf2.70265)
Supplement: Supplementary file 1 [file PMF2-2-e70265-s001.docx]

Table S1. Clinical associations with kidney disease stratified by race.

| **Characteristic** | **Black**  N = 38^1^ | **Non-black**  N = 65^1^ | **p-value**^2^ |
| --- | --- | --- | --- |
| **Maternal age at delivery (years)** | 33 (28, 37) | 30 (26, 35) | 0.2 |
| **GA at entry to care** | 9.0 (7.6, 12.6) | 8.5 (6.4, 10.3) | 0.2 |
| **Body mass index (kg/m^2)** | 29 (25, 35) | 27 (22, 32) | 0.11 |
| **Nulliparous** | 12 (32%) | 28 (43%) | 0.2 |
| **Pre-pregnancy renal disease diagnosis** | 27 (71%) | 55 (85%) | 0.10 |
| **Pre-pregnancy hypertension** | 36 (95%) | 41 (63%) | <0.001 |
| **Pre-pregnancy diabetes** | 14 (37%) | 15 (23%) | 0.13 |
| **Renal transplant before pregnancy** | 2 (5.3%) | 9 (14%) | 0.2 |
| **Any dialysis before, during, or after pregnancy** | 19 (50%) | 26 (40%) | 0.3 |
| **Worsening renal function during pregnancy** | 19 (50%) | 35 (54%) | 0.7 |
| **Hypertensive disorders of pregnancy** | 23 (61%) | 32 (49%) | 0.3 |
| **Genetic counseling referral for any reason** | 25 (66%) | 47 (72%) | 0.5 |
| **Genetic counseling referral for renal disease** | 5 (13%) | 13 (20%) | 0.4 |
| **Did the GC visit happen** | 21 (84%) | 42 (88%) | 0.7 |
| **Did the GC visit address renal disease** | 2 (9.1%) | 13 (31%) | 0.050 |
| **Was there ever a recommendation for renal genetic testing** | 6 (16%) | 13 (20%) | 0.6 |
| **Did the patient undergo renal genetic testing** | 1 (17%) | 7 (54%) | 0.2 |
| ^1^Median (Q1, Q3); n (%) | | | |
| ^2^Wilcoxon rank sum test; Pearson's Chi-squared test; Fisher's exact test | | | |

Table S2. Clinical associations with kidney disease stratified by insurance.

| **Characteristic** | **Commercial**  N = 44^1^ | **Medicaid/Medicare/CHIP/Self-Pay**  N = 59^1^ | **p-value**^2^ |
| --- | --- | --- | --- |
| **Maternal age at delivery (years)** | 31 (27, 35) | 32 (26, 37) | 0.5 |
| **GA at entry to care** | 7.6 (6.2, 8.8) | 10.0 (8.2, 12.6) | <0.001 |
| **Body mass index (kg/m^2)** | 28 (22, 34) | 28 (23, 34) | 0.7 |
| **Nulliparous** | 25 (57%) | 15 (25%) | 0.001 |
| **Pre-pregnancy renal disease diagnosis** | 38 (86%) | 44 (75%) | 0.14 |
| **Pre-pregnancy hypertension** | 29 (66%) | 48 (81%) | 0.074 |
| **Pre-pregnancy diabetes** | 9 (20%) | 20 (34%) | 0.13 |
| **Renal transplant before pregnancy** | 3 (6.8%) | 8 (14%) | 0.3 |
| **Any dialysis before, during, or after pregnancy** | 15 (34%) | 30 (51%) | 0.090 |
| **Worsening renal function during pregnancy** | 20 (45%) | 34 (58%) | 0.2 |
| **Hypertensive disorders of pregnancy** | 23 (52%) | 32 (54%) | 0.8 |
| **Genetic counseling referral for any reason** | 32 (73%) | 40 (68%) | 0.6 |
| **Genetic counseling referral for renal disease** | 7 (16%) | 11 (19%) | 0.7 |
| **Did the GC visit happen** | 28 (88%) | 35 (85%) | >0.9 |
| **Did the GC visit address renal disease** | 7 (24%) | 8 (23%) | >0.9 |
| **Was there ever a recommendation for renal genetic testing** | 9 (20%) | 10 (17%) | 0.7 |
| **Did the patient undergo renal genetic testing** | 7 (78%) | 1 (10%) | 0.005 |
| ^1^Median (Q1, Q3); n (%) | | | |
| ^2^Wilcoxon rank sum test; Pearson's Chi-squared test; Fisher's exact test | | | |
